# Airborne Fungal Monitoring in Healthcare Environments: A Systematic Review

**DOI:** 10.3390/jof12050336

**Published:** 2026-05-04

**Authors:** Dana L. Surwill, Patricia Cruz, Mark P. Buttner, Jennifer R. Pharr, Nancy Lough, Theresa T. Roehr

**Affiliations:** 1 Department of Environmental and Global Health, School of Public Health, University of Nevada, Las Vegas, NV 89154, USA; surwill@unlv.nevada.edu (D.L.S.);; 2 Department of Educational Psychology, Leadership, and Higher Education, College of Education, University of Nevada, Las Vegas, NV 89154, USA

**Keywords:** fungi, airborne pathogens, monitoring, surveillance, healthcare facilities, monitoring guidelines, indoor air sampling, *Aspergillus*, *Penicillium*, *Alternaria*

## Abstract

Background: Fungal infections pose a significant threat to public health, with over 6.55 million cases and 2.55 million deaths annually. Exposure to fungal spores in indoor environments primarily occurs through inhalation or direct contact with surfaces. Monitoring is critical for early detection and prevention of outbreaks, yet routine airborne fungal testing is not universally mandated across healthcare settings. Methods: A systematic review of peer-reviewed articles from four databases was conducted to identify current airborne fungal monitoring guidelines and best practices for sample collection, culture media, incubation conditions, and results interpretation. Results: Eighteen articles met the inclusion criteria, and four studies discussed potential guidelines for acceptable airborne fungal levels in healthcare environments. Guidelines ranged from <1 CFU/m^3^ for HEPA-filtered environments to >1000 CFU/m^3^ for non-filtered areas. The most common fungi identified were *Aspergillus*, *Penicillium*, *Alternaria*, *Cladosporium*, and *Rhizopus*, with six WHO-listed critical fungal pathogens found. Impaction was the sole sampling method used, with most studies employing Sabouraud dextrose or malt extract agar with chloramphenicol, incubation for 2–7 days at 25–30 °C, and morphological identification. Conclusions: The need for globally recognized fungal monitoring standards is pressing. Without them, preventable fungal exposure will persist, risking severe, potentially fatal infections for patients and healthcare workers.

## 1. Introduction

Each year, over 6.55 million individuals globally are affected by life-threatening fungal infections, leading to approximately 2.55 million deaths directly attributable to fungal diseases [1]. In the United States alone, 666,235 individuals were diagnosed with a fungal infection during inpatient hospital stays in 2018, leading to a $6.7 billion burden on the healthcare system [2]. Furthermore, 7199 people in the U.S. died from a fungal infection in 2021 [3]. This alarming trend highlights the need for increased awareness and action within the healthcare community. Despite advancements in public health measures, these numbers continue to rise, placing both patients and hospital staff at an increased risk of severe disease.

Infections caused by various fungal species are a major concern in healthcare settings. Fungi, including molds and yeasts, are eukaryotic microorganisms that can cause superficial, cutaneous, subcutaneous, mucosal, and systemic diseases [4]. Although many fungi are nonpathogenic and reside naturally in the human body, others can be highly infectious, especially when invasive. Airborne fungi commonly found in indoor and outdoor environments are primarily spread through inhalation of spores or direct contact with spores on surfaces [4]. The risk of fungal exposure in healthcare environments increases when high humidity or moisture levels that promote fungal growth are present and are often due to building damage or renovations [5].

Healthcare facilities, such as hospitals, clinics, and emergency departments, are critical environments for fungal monitoring, as they house many individuals with compromised immune systems, including chemotherapy patients, organ transplant recipients, and those with chronic diseases. Environmental conditions, such as humidity, temperature, and surface type, contribute to fungal proliferation, with damp areas often fostering mold growth. When aerosolization of fungal spores occurs, fungi can be inhaled, especially in ventilated spaces where molds are prone to grow. Showers and water taps can also facilitate the transmission of airborne fungal spores [6]. Immunocompromised patients are particularly vulnerable to opportunistic fungal infections. Furthermore, invasive medical procedures, such as surgeries and catheterizations, provide pathways for fungi to enter the body, increasing infection risks [7]. Inadequate cleaning, disinfection practices, and insufficient ventilation can exacerbate fungal outbreaks in these settings.

*Aspergillus*, *Pneumocystis*, and *Candida* are among the most common fungal pathogens identified in healthcare environments. These three genera are responsible for 76% of diagnosed fungal infections and over 81% of fungal-related costs [2]. *Aspergillus* species, notably *Aspergillus fumigatus*, can lead to invasive aspergillosis, which is particularly concerning for immunocompromised patients, those who have undergone major surgeries, and individuals in burn units [8,9]. *Pneumocystis jirovecii* (previously classified as *Pneumocystis carinii*) is another concerning pathogen, causing severe pneumonia, especially in individuals with HIV/AIDS or other immunocompromising conditions [10]. Infections from *Candida* can result in long-term recovery, costly treatment, and high mortality [11]. The rise in antifungal resistance, particularly in *Candida* species, complicates treatment strategies, heightening the risk of outbreaks within healthcare environments [12]. *Candidozyma auris*, formerly classified as *Candida auris*, is an emerging multidrug-resistant organism that is not killed using standard cleaning practices and is increasingly problematic due to its ability to colonize patients for multiple months [13]. Additionally, the potential for biofilm formation on medical devices and surfaces provides a persistent reservoir that can facilitate infection and further complicate the eradication of these pathogens [14].

Although antifungal medications are commonly used to treat superficial and systemic fungal infections, resistance to these treatments is becoming a growing concern. This resistance complicates infection management, leading to prolonged illness, higher mortality, and increased healthcare costs. As traditional antifungal agents lose their efficacy, new therapeutic approaches are needed. Therefore, it is crucial to reduce fungal infection rates through enhanced infection control measures, hygiene education, and environmental management to mitigate the strain on healthcare systems.

Routine fungal monitoring is critical for identifying pathogens, assessing overall cleanliness, and implementing timely infection control measures in healthcare facilities, especially for immunocompromised patients who are at a heightened risk of infection [2]. However, there is currently no universal requirement for routine fungal surveillance in healthcare facilities. The World Health Organization (WHO) recommends enhancing surveillance capacity due to the rise in antifungal resistance [15]. Some countries have monitoring practices in place for specific healthcare areas, such as intensive care units, bone marrow transplantation rooms, and tissue and cell banks [16,17]. Systematic fungal monitoring, particularly airborne sampling, could serve as a proactive strategy to enhance infection control measures, improve patient safety, and ensure continuous surveillance in healthcare settings.

Fungal monitoring methods, including air sampling, settle/gravity plates, and surface sampling, offer distinct advantages and limitations. Air sampling, essential for assessing airborne fungal contamination, is crucial in environments, such as hospitals, where inhalation is a significant risk. While air sampling can provide a broad overview of fungal presence, it often requires specialized equipment and may not capture all fungal species due to variations in airflow, sampling duration, and culture media [18]. Settle plates provide a simpler and more cost-effective means of monitoring fungal contamination; however, the results are nonquantitative and nonrepresentative. Therefore, this sampling method is not recommended [19]. Surface sampling targets specific areas where fungal contamination is likely to be present, such as high-touch surfaces and equipment, allowing for precise identification of localized fungi [20]. While this method is relatively easy to perform and is inexpensive, it can overlook airborne fungi and is reliant on proper sampling and analysis techniques for accurate results. Each method’s efficacy can greatly depend on the specific circumstances and goals of the analysis.

The objective of this study was to identify airborne fungal monitoring guidelines and review fungal prevalence by location and type of healthcare facility. In addition, the review aimed to determine the best practices for airborne fungal monitoring in healthcare environments, including sample collection, selection of culture media, incubation conditions, and results interpretation for various fungal pathogens. By implementing routine fungal monitoring, healthcare facilities can enhance their ability to detect and mitigate the risks posed by airborne fungi.

## 2. Materials and Methods

The checklist provided by Preferred Reporting Items for Systematic Review and Meta-Analysis (PRISMA) was utilized to conduct a literature review of peer-reviewed articles. The Joanna Briggs Institute (JBI) guidelines were utilized for quality assessment. The articles identified from the literature review were grouped for synthesis based on the healthcare facility and type of air sampling method.

### 2.1. Search Strategy

Research articles were identified from four electronic databases: Google Scholar, PubMed, Scopus, and Web of Science. The search string used was indoor air sampling or monitoring guidelines AND *Candida* or *Aspergillus* or *Cryptococcus* AND hospital or clinic or healthcare facility. Articles published between January 2020 and December 2024 were selected due to increased airborne pathogen monitoring during and after the SARS-CoV-2 pandemic. The abstracts of articles were screened for duplication, inclusion criteria, and exclusion criteria before the identified articles were reviewed. The full text of articles meeting the inclusion criteria were gathered, examined, and synthesized in the results of this study.

### 2.2. Inclusion and Exclusion Criteria

Articles were included based on the following criteria: (1) written in English, (2) published between January 2020 and December 2024, (3) utilized an air sampling method, (4) identified a type of fungus, and (5) conducted inside of a healthcare facility. Articles were excluded if they were written in a language besides English, collected only surface samples, did not collect samples of fungi, or collected samples outside of a healthcare facility (e.g., homes, apartments).

### 2.3. Quality Assessment

The Joanna Briggs Institute (JBI) checklists for cross-sectional studies [21], quasi-experimental studies [22], and cohort studies [23] were used to assess the risk of bias in the selected studies. The tool helps evaluate the study quality based on its methodology and outcomes. Two independent reviewers conducted the assessment, and each study was scored using the following system: Yes = 2; No = 0; Unclear = 1. Questions that were not applicable (N/A) were not scored. Total possible points and scores for risk of bias were adjusted to accommodate the N/A answers.

## 3. Results

### 3.1. Search Results

**Figure 1:**
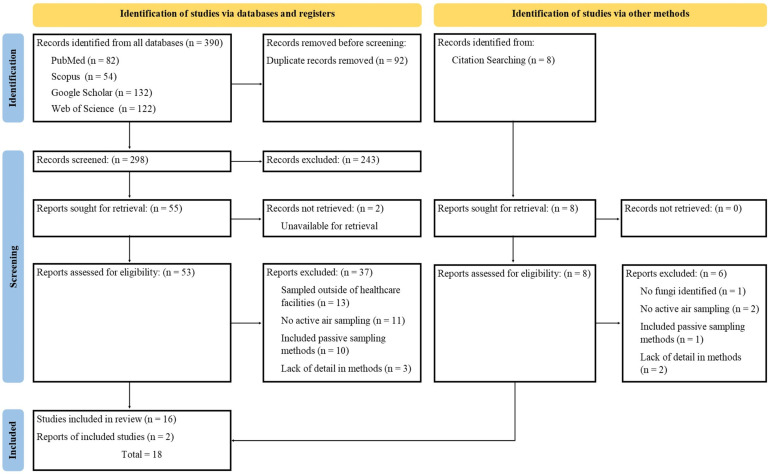
Preferred Reporting Items for Systematic Reviews and Meta-Analyses (PRISMA) flow diagram summarizing the screened, included, and excluded data.

A total of 390 articles were identified from the four databases and after the removal of duplicates, 298 articles remained (Figure 1). The titles and abstracts were screened and evaluated using the inclusion and exclusion criteria. A total of 243 articles were removed for not meeting the inclusion criteria, and 55 articles were further assessed for eligibility. Thirty-seven of the 55 were excluded for not collecting air samples, including passive sampling methods, collecting samples outside of a healthcare facility, and having unclear methodology. A total of 16 articles were selected for inclusion, and additional studies were identified through citation searching. Eight articles were screened through citation searching, and two were selected for inclusion. Therefore, a total of 18 articles were included in this review. The JBI critical appraisal tool was employed to assess the quality of all included studies. The number of checklist questions varied according to study design: cross-sectional studies (Q1–Q8), cohort studies (Q1–Q11), and quasi-experimental studies (Q1–Q9). Total scores were determined based on the number of applicable questions. Across study types, the risk of bias was generally low to moderate, with 17 studies rated as low risk and the one study from Buchanan et al. (2020) [24] as moderate risk (Table A1, Table A2 and Table A3). Notably, none of the included studies was assessed as high risk. As a result, all studies met the methodological quality criteria and were included in the final synthesis.

### 3.2. Geographic Distribution

The 18 studies included in this review were conducted in 13 countries (Table 1). Of these, four studies originated from Iran and three studies were conducted in Brazil. The remaining countries represented by the studies were Burkina Faso, China, Cyprus, Indonesia, Japan, the Philippines, Poland, Saudi Arabia, Taiwan, the United Kingdom, and the United States. Climatic factors were not systematically extracted and reported. Based on the local climate, some of the healthcare facilities did not employ climate control measures (e.g., air conditioning) throughout the entire facility [25].

### 3.3. Sampling Location

All included studies sampled healthcare facilities. However, there were many different areas of healthcare facilities sampled (Table 1). Buchanan et al. (2020) [24] sampled the indoor air in hospital wards using mobile dust-containment carts which are used to minimize air contamination from construction, while others targeted critical care areas, such as the ICU [34] and hematology wards [39]. Sampling was also performed in specific hospital zones, such as gynecology, cardiology, and internal medicine departments [32], as well as in specialized settings, such as air-conditioned versus non-air-conditioned wards [25].

At least one fungal species [29,32,40] and as many as 21 different fungi [38] were detected in the included studies (Table 1). The highest reported total fungal concentration was in a pediatric hematology ward (345 CFU/m^3^) [31] (Table 2). Three other studies reported fungal concentrations over 200 CFU/m^3^ in hematology, lung, transplant, intensive, medical, and emergency wards [27,31,34,41]. Operating rooms and intensive care units in six of the studies reported mean fungal concentrations below 56 CFU/m^3^ [27,33,35,36,41]. Two of the studies examined the effect of HEPA filtration on airborne fungal pathogens [24,33]. Both studies saw a reduction in fungal concentrations after utilizing HEPA filters. However, one study found a lower average fungal concentration in a non-air-conditioned ward (73 CFU/m^3^) when compared to an air-conditioned ward (151 CFU/m^3^) within the same hospital [25].

### 3.4. Culture Media

Various culture media were employed across studies for fungal isolation (Table 3). Malachite green agar [29], dichloran rose-bengal chloramphenicol agar [30,34], Sabouraud dextrose agar [26,27,31,33,36,38,40], and malt extract agar [25,35,39,41] were the most common. However, four studies utilized other types of agars. One used glass microfiber filter media [28] and another used Columbia blood agar with 5% blood [32]. The final two used potato dextrose agar [37] and inhibitory mold agar [24]. Eleven of the 18 studies utilized agar plates that were supplemented with chloramphenicol to suppress bacterial growth.

### 3.5. Sampler Type, Flow Rate, and Sampling Time

All of the studies included in the review employed impaction as the active air sampling technique (Table 3). However, many different types of samplers were used, with the most common being the Andersen single-stage sampler [30,33,35,38,41]. Ablola and Bungay (2020) [25] used an Andersen six-stage sampler and other studies used samplers, such as the MAS-100NT Microbial Air Sampler, that have a higher flow rate [27].

Six studies did not report the height of the sampler from the floor or the distance from the wall and obstacles. However, the other 12 articles all stated that the sampler was placed between 1 and 1.5 m from the floor, and some studies stated that the sampler was placed at least 1 m from walls and obstacles [27,28,29,30,31,33,34,35,38,39,40,41]. A few studies placed the samplers in the middle of the assessed rooms.

The most common sampling flow rate was 28.3 L/min or 100 L/min, depending on the sampler used. The total volume of air collected ranged from 50 to 1000 L per sample. While some studies only reported the total volume of air collected, others collected samples for a specified amount of time. In these studies, the air was sampled between 5 and 15 min.

### 3.6. Incubation Time/Temperature

Incubation conditions varied based on the fungal species targeted and the culture media used in each study (Table 3). Most studies performed incubation at 25 to 30 °C for 2 to 7 days. Some studies incubated the samples for up to 10 days for necessary fungal growth confirmation [27,31,33]. However, two studies incubated samples at 37 °C for 2 to 5 days [25,32] and Ketabi et al. (2022) incubated samples at 32 °C for 7 to 10 days [33].

### 3.7. Identification Methods

Various analysis methods were used in the studies reviewed (Table 3). The most common practice was using macroscopic and microscopic morphologic characteristics to identify the fungal isolates. Multiple studies stated that the cost of more advanced methods, such as polymerase chain reaction (PCR) or mass spectrometry, prevented more accurate analysis. By examining the morphological characteristics, such as colony appearance, spore structures, and microscopic features, most fungal pathogens were identified. The fungal isolates that were unknown in these studies were reported as “other fungi”. Chen et al. (2024) utilized PCR to identify the fungal pathogens present while two additional studies utilized PCR to more accurately identify the fungal species [28,30,34]. However, Lemos et al. (2024) only used the PCR technique on a few select samples due to the cost of analysis [34].

### 3.8. Fungal Prevalence

**Figure 2:**
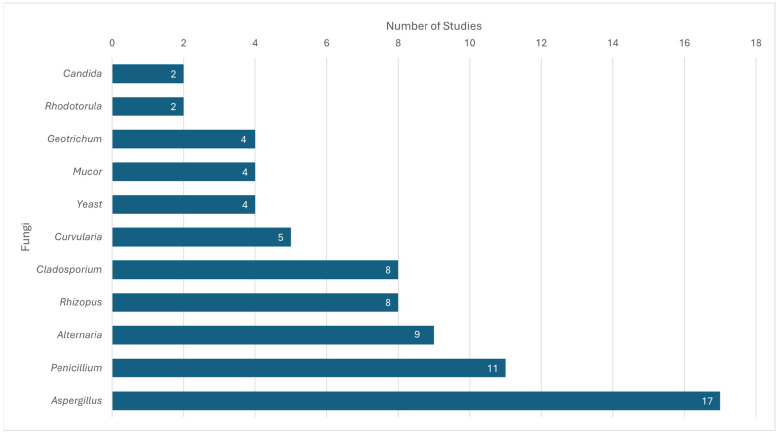
Fungal prevalence in the included studies.

*Aspergillus* species were the most prevalent airborne fungi identified in healthcare environments, as they were detected in all but one study (Table 1 and Figure 2). Additionally, thirteen *Aspergillus* species were named within the studies, with the most prominent being *A. fumigatus*, *A. niger*, and *A. flavus*. Other notable fungal genera included *Penicillium*, which was reported in eleven studies, and *Alternaria* and *Rhizopus*, which appeared in nine and eight studies, respectively [24,25,27,28,31,33,35,36,37,39,41]. Additionally, *Cladosporium* was cultured in eight studies [24,31,35,36,37,41]. Various other fungal pathogens were found in the included studies, such as *Candida*, *Fusarium*, and *Trichoderma* [30,35].

Five studies reported concentrations for individual fungi along with or instead of total fungal concentrations (Table 4). *Aspergillus* spp. and *A. fumigatus* were the most commonly reported, with concentrations of 0–89 and 0–255 CFU/m^3^, respectively [25,27,31,34,40]. In one study, *A. fumigatus* concentrations were higher in air-conditioned rooms (151 CFU/m^3^) when compared to non-air-conditioned rooms (73 CFU/m^3^) [25]. *Aspergillus flavus* (0–53 CFU/m^3^), *A. niger* (18–49 CFU/m^3^), and *Rhizopus* (1–14 CFU/m^3^) concentrations were only reported in one of the five studies [25]. The concentrations of *Penicillium* reported in three studies ranged from 0 to 86 CFU/m^3^ [25,27,31]. *Alternaria* had lower concentrations reported in two studies with fungal counts of 0 to 10 CFU/m^3^ [25,31]. Only one study reported *Cladosporium* with concentrations of 0 to 131 CFU/m^3^ [31].

Six studies collected outdoor samples to analyze outdoor-to-indoor effects of fungal prevalence. Several studies found that outdoor fungal concentrations were higher than most indoor areas. Alghamdi et al. (2023) reported total fungal concentrations much higher outdoors (25–260 CFU/m^3^) than in indoor rooms (0–18 CFU/m^3^) [26]. Van Rhijn et al. (2021) similarly observed a statistically significant difference in *A. fumigatus* concentrations, with indoor counts generally lower than outdoor counts, reporting an average indoor concentration of 3 ± 5 CFU versus 16 ± 25 CFU outdoors [39]. In contrast, other studies found that certain indoor areas exceeded outdoor levels. Montazeri et al. (2020) reported an outdoor concentration of 40 ± 23 CFU/m^3^, with only the operating room (32 ± 31 CFU/m^3^) falling below outdoor levels, while all other wards exceeded them [36]. Yousefzadeh et al. (2022) similarly identified an outdoor concentration of 53 CFU/m^3^, with only the operating room (0.42 CFU/m^3^) and burn unit (43 CFU/m^3^) registering lower concentrations than outside, while all other wards exceeded outdoor levels [41]. In another study, the authors found no difference in the presence of indoor and outdoor airborne fungi, reporting an outdoor concentration of 151 ± 111 CFU/m^3^, with only the NICU and surgical centers testing lower [38]. Nascimento et al. (2023) likewise concluded that outdoor air did not influence the concentration of airborne yeast in the analyzed environments [30].

Regarding fungal species and genera, findings were also mixed. Montazeri et al. (2020) reported that *Penicillium* was the most frequently isolated genus indoors, while *Alternaria* was the most abundant outdoors, though almost all studied species were detected in both indoor and outdoor air samples [36]. Another study that reported no indoor-outdoor difference similarly found no distinction in the most frequently detected fungal genera between settings [38].

### 3.9. Guidelines

No universal guidelines for fungal concentrations in healthcare facilities were found. However, many studies reported various recommendations made by national or international agencies. Four of the 18 studies mentioned a potential guideline to determine acceptable airborne fungal levels in indoor healthcare environments (Table 1). These guidelines vary widely, with some suggesting that fungal concentrations for *Aspergillus* spp. should be maintained below 1 CFU/m^3^ in environments equipped with high-efficiency particulate air (HEPA) filtration or 5 CFU/m^3^ without HEPA filtration, while others set thresholds for total fungal concentrations up to 750 CFU/m^3^ [25,34]. Additional recommendations for bioaerosols generally advise keeping indoor fungal levels below 1000 CFU/m^3^ to minimize infection risks [32,36]. The recommended guideline from the WHO is less than 50 CFU/m^3^ for airborne fungi in healthcare facilities [36]. Additionally, the European Commission Report developed recommendations for bioaerosols. It determined concentrations of 0 CFU/m^3^ were undetectable, 1–499 CFU/m^3^ were low, 400–999 CFU/m^3^ were medium, and over 1000 CFU/m^3^ were high [32,36]. None of the studies recommended a guideline for individual fungal pathogens or specific hospital areas.

## 4. Discussion

Fungal monitoring is essential for early detection and prevention of fungal proliferation in healthcare environments, yet routine testing is not mandated. Therefore, it is imperative to understand the current practices related to sampling, analysis, and interpretation of results. This systematic literature review revealed that only four of the 18 studies mentioned specific guidelines or recommendations to determine acceptable airborne fungal levels in indoor healthcare environments. This review also summarized best practices for sample collection, culture media, and incubation conditions for fungi. Because so many options are available in terms of air samplers and culture conditions for the detection of fungi, analysis of current methodology in healthcare settings can inform infection control practices.

### 4.1. Sampling Methodology

Proper selection and deployment of samplers play critical roles in accurately assessing airborne fungal contamination within healthcare settings. All reviewed studies employed impaction sampling. Other types of sampling include settle plates and surface sampling, but settle plates are nonquantitative and nonrepresentative, and surface sampling can overlook airborne fungi present at the time of sampling [19]. The use of surface sampling alongside air sampling can provide details about microbial sources as well as a more comprehensive understanding of the fungi present [19]. The most common placement of samplers identified in our study is in agreement with previous research recommending the use of impaction samplers placed at the height of the patient breathing zone, approximately 1.5 m above the floor [42]. Additionally, Tomazin and Matos (2024) suggest ensuring the HVAC system is powered on during sampling to provide optimal results [42].

Flow rates and sampler duration reported in this review varied. The flow rate is generally set by the type of impactor used; however, the total volume of air and the sampling duration are more important for culture analysis. A recent study states that samplers should not collect more than 1000 L or approximately 10 min to prevent spore desiccation and culture media dehydration which can compromise fungal growth [42].

Various culture media were used across the studies to isolate fungi. The use of SDA with chloramphenicol has been shown as a reasonably consistent and sensitive medium to utilize for fungal sampling [43]. The other media included in the various studies were each chosen for their specific properties in promoting the growth of selected fungal species. The use of antimicrobials, such as chloramphenicol, suppressed bacterial contamination, enhancing the accuracy of fungal identification [44]. Choosing the correct type of agar plays a crucial role in the successful cultivation and differentiation of fungi. Therefore, it is recommended to consider target fungi when selecting culture media.

Incubation times and temperatures varied depending on the targeted fungal species and culture media. While environmental fungi, yeasts, *Rhizopus*, *Aspergillus niger*, and *Fusarium* prefer growth at 25 °C, *A. fumigatus* grows best at warmer temperatures, between 35 and 40 °C [45]. *Aspergillus fumigatus* can lead to invasive aspergillosis, which is particularly concerning for immunocompromised patients, those who have undergone major surgeries, and individuals in burn units [8,9]. Therefore, it is recommended to collect and incubate duplicate samples with one at 25 °C for 3 to 5 days and the other at 37 °C for 2 days (Table 5) and outdoor reference samples [19,45]. This would ensure optimal growth for most fungal isolates as well as for *A. fumigatus*, which is on the WHO fungal pathogen priority list [15].

The choice of sampling location within the analyzed studies was tailored to target areas of increased infection risk for patients. Important fungal testing areas in healthcare facilities include general patient areas, immunocompromised patient areas, and rooms or corridors impacted by construction [46,47]. The U.S. Occupational Safety and Health Administration (OSHA) recommends that air ducts and ventilation filters should be cleaned and replaced on a routine schedule to prevent unintentional dispersal of fungal pathogens [48]. Additionally, areas under construction or renovation should be isolated by plywood or polyethylene sheeting and employ negative pressure ventilation with HEPA filtration to ensure contamination of other areas does not occur. Sampling should also occur before and after any construction or remodeling project, regardless of location within the facility [48]. These methodological considerations, including sampler type, placement height, flow rate, and sampling duration, significantly influence the detection sensitivity and reliability of fungal counts. Without the proper sampling methodology for healthcare facilities, fungi may be incorrectly identified, proper disinfection practices cannot be ensured, and infection control strategies may be ineffective. By carefully selecting sampling locations and implementing proper environmental controls, healthcare facilities can significantly reduce the risk of fungal contamination and protect staff and vulnerable patient populations.

### 4.2. Fungal Identification

Three of the most common fungal pathogens identified in healthcare environments are *Aspergillus*, *Pneumocystis*, and *Candida* [2]. *Aspergillus* was reported in 17 of the 18 studies included in this review, matching the previously reported fungal prevalence [2]. However, the studies analyzed did not recover airborne *Pneumocystis*, likely because it does not grow on routine microbiological media [49]. *Candidozyma auris* was also not reported, but several studies reported yeasts or *Candida* spp., which were not speciated. The most commonly found fungi in the 18 included studies were *Aspergillus*, *Penicillium*, *Alternaria*, *Cladosporium*, and *Rhizopus*. In 2022, the WHO published the first global fungal pathogens priority list, which prioritizes pathogens in terms of public health importance [15]. Six of the pathogens identified in this review (i.e., *Cryptococcus*, *Aspergillus fumigatus*, *Candida glabrata*, *Fusarium*, *Candida parapsilosis*, and *Mucorales* [a complex taxonomic fungal group, including eleven different genera that can infect humans]) are on the WHO priority list [50]. These pathogens pose significant risks to vulnerable populations, such as those with weakened immune systems, undergoing surgeries, or in critical care. Of more concern, these pathogens can persist in the air, on surfaces, and on medical equipment as they are resistant to standard cleaning products [13]. Once infected, patients may experience long and costly treatments, furthering the risk of complications and death.

The identification of fungi in the included studies predominantly relied on morphological characteristics, which, although cost-effective and accessible, have limitations. This approach, which relies on microscopic and macroscopic features, is often time-consuming, requires significant expertise, and may lack accuracy due to overlapping characteristics among different fungal species [51]. There was limited utilization of molecular techniques, such as polymerase chain reaction (PCR) and Matrix-Assisted Laser Desorption/Ionization Time-of-Flight (MALDI-ToF) mass spectroscopy in most studies due to the cost associated with these types of analysis. PCR is a molecular technique used to amplify specific DNA sequences, making it faster and more sensitive than culture to identify pathogens. MALDI-ToF is a method that rapidly identifies microorganisms by analyzing their unique protein fingerprints, providing fast and accurate species-level identification of fungi and also antifungal resistance [52,53,54]. Integrating PCR-based methods, MALDI-ToF, or other enhanced molecular techniques into routine surveillance could significantly improve detection accuracy, especially for difficult to culture fungi, emerging pathogens, and resistant species [55]. Multidrug-resistant *Aspergillus fumigatus*, *Alternaria*, *Cladosporium*, and *Rhizopus* were the most prominent fungi reported in two of the studies [56,57]. The recovery of these fungi, coupled with their rising drug-resistance, demonstrates the need for accurate and rapid detection strategies [58].

Molecular techniques are not without their limitations. For example, in one study that employed both PCR and culture techniques, culture analysis was only 56% sensitive, whereas PCR was successful in detecting 89% of fungal DNA when histopathological reports were used as a reference [59]. However, because culture analysis was less sensitive, it could not serve as a reliable reference to confirm the species identified by PCR. As a result, there was no definitive way to verify the accuracy of the PCR-based species identification, highlighting a limitation in relying solely on culture as a gold standard [53]. While MALDI-ToF is a promising tool, it requires a comprehensive database that may not detect very similar or emerging fungal pathogens [60]. It also requires proper technical preparation and a sufficient amount of biomass to report proper results, which necessitates advanced training. While these enhanced methods have limitations, their integration into routine surveillance is particularly vital for the rapid detection of multidrug-resistant fungi [58].

### 4.3. Environmental Factors

Humidity, temperature, and ventilation play a pivotal role in fungal proliferation and aerosolization. Addressing these variables is critical, as damp conditions, water leaks, and poor ventilation are well-known contributors to fungal growth [48]. The studies in this review came from geographically diverse locations, which face different environmental factors that influence fungal pathogen presence. Based on the local climate, some of the healthcare facilities do not employ climate control measures (e.g., air conditioning) in all of the facility, which can influence fungal proliferation. Previous research has shown that the microbial load in naturally ventilated areas of public hospitals was significantly higher than that of air-conditioned areas [61]. Utilizing HEPA filtration significantly decreased the transmission of airborne fungi and the risk of infection [61]. However, if not properly maintained and cleaned, HVAC systems can increase the risk of exposure. Therefore, understanding and managing these environmental factors through regular maintenance and appropriate climate control strategies are essential steps in minimizing fungal contamination and protecting patient health across diverse geographic settings.

### 4.4. Guidelines

Four of the 18 studies identified guidelines/recommendations for total fungal load. Guidelines varied widely, ranging from <1 CFU/m^3^ for HEPA filtered environments to >1000 CFU/m^3^ for non-filtered areas. Unfortunately, it was difficult to ascertain which thresholds are supported by stronger evidence. Therefore, the guidelines should not be interpreted as equivalent. In summary, the WHO threshold of less than 50 CFU/m^3^ for airborne fungi in healthcare facilities represents a recommendation with broader organizational or multi-study backing [36]. In contrast, the threshold of <1 CFU/m^3^ for HEPA-filtered environments and <5 CFU/m^3^ for non-filtered areas originates from a single study [25], and the <750 CFU/m^3^ limit reflects a national standard from Brazil’s Ministry of Health [34] that may not be generalizable across healthcare contexts. The European Commission’s bioaerosol categorization, classifying concentrations of 1–499 CFU/m^3^ as low, 500–999 CFU/m^3^ as medium, and >1000 CFU/m^3^ as high, provides a broader contamination framework, but was not developed specifically for healthcare environments [32,36]. Without standardized protocols, under- or over- interpreting CFU values can cause unjustified safety or alarm. Under-interpreting could result in an increased level of preventable infection incidence, whereas over-interpreting could cause unnecessary costly treatment or cleaning to be implemented. For example, a ward detecting fungi at a concentration of 60 CFU/m^3^ would fall within the European Commission’s “low” category, yet exceed the WHO’s recommended threshold, leaving infection control staff without a clear, consistent basis for action. Without a universally accepted guideline for total airborne fungal contamination in healthcare settings, it is difficult to implement consistent infection control measures. Standardized guidelines will help reduce the variability in fungal contamination assessment, ensuring more consistent, accurate, and reliable monitoring. The analyzed studies did not report any guidelines for specific fungal pathogens, but previous studies have estimated that 100 *Alternaria* spores and 3000 *Cladosporium* spores per m^3^ can evoke allergic symptoms when using a spore trap such as the Burkard sampler [62]. Additionally, *Aspergillus* concentrations higher than 50 CFU/m^3^ have shown a potential association with sick-building syndrome, which can cause allergies, headaches, nausea, and fatigue associated with time spent in a specific building [62]. *A. fumigatus* is generally considered low risk if its concentration is lower than 10 CFU/m^3^ [63]. Because of the concern for opportunistic pathogens, targeting fungal species, such as *Aspergillus fumigatus*, should take priority when designing an infection control program. In addition to specific fungal pathogen loads, concentrations by area of a hospital should also be considered when developing universal guidelines. Protective areas such as ICUs, burn units, and operating rooms may benefit from stricter guidelines, whereas general hospital areas may not need as strict of guidelines. Developing internationally recognized guidelines, supported by comprehensive research, is essential to define actionable thresholds that can inform environmental management strategies.

### 4.5. Limitations

This review was restricted to articles published in English, which may have resulted in overlooking critical findings from non-English studies. The use of only four search engines to gather data may have constrained the diversity of information retrieved and may have caused relevant studies to be missed. Additionally, by excluding surface sampling and other environmental assessment methods, this review may have overlooked critical sources of fungal spores, resulting in an incomplete understanding of fungal contamination within healthcare environments. Finally, the diverse environmental and climatic conditions across study locations influenced fungal prevalence, but these factors were not quantitatively addressed, limiting the generalizability of the findings in this study.

While there were limitations, this study had several strengths. This systematic review provides a comprehensive synthesis of recent peer-reviewed literature, focusing on airborne fungal monitoring in diverse healthcare settings across multiple countries. The application of the PRISMA methodology ensures a transparent and structured approach to literature selection. Additionally, the review thoroughly analyzed various aspects of sampling methodologies, including sampler types, culture media, incubation conditions, and identification techniques, which offer valuable insights into best practices and current limitations in healthcare facilities. By highlighting the prevalence of common fungi and emphasizing the lack of standardized international guidelines, this study identified critical gaps in infection control protocols. This review advances the understanding of fungal contamination sampling and helps to inform the need for developing standardized, effective monitoring strategies to improve patient and staff safety in healthcare environments.

### 4.6. Recommendations

There is a need for a multidisciplinary approach to fungal surveillance in healthcare facilities. Stringent recommendations for total and/or individual fungal concentrations in healthcare facilities by clinical area should be developed by international agencies while infection control staff, microbiologists, and hospital administration conduct routine fungal monitoring, apply standardized guidelines, and respond proactively to fungal contamination. This should include implementing effective cleaning and disinfection practices and ensuring ventilation systems are properly installed and maintained, to prevent fungal proliferation. Additionally, educating and training personnel on best practices for sampling, analysis, and results interpretation are equally important to ensure data accuracy and actionable results. Air sampling should be conducted alongside surface sampling and outdoor sampling to provide a full picture of the fungi present, and PCR should be used to identify difficult to culture fungi, increasing the accuracy in fungal identification. Given that outdoor fungal concentrations frequently exceed indoor levels in certain ward types but not others, outdoor sampling should be incorporated into routine surveillance protocols to better contextualize indoor counts and identify infiltration risk by clinical area. Enhancing infection control measures and promoting prevention education among staff, patients, and visitors are vital strategies to mitigate infectious disease outbreaks and reduce infection rates, thereby alleviating the strain on healthcare systems.

Future research should focus on developing standardized, cost-effective protocols for fungal sampling and analysis that can be widely adopted across healthcare settings. This includes establishing universally accepted guidelines for permissible fungal concentrations, tailored to high-risk patient areas. Additionally, there is a pressing need to integrate enhanced detection methods, such as PCR and mass spectrometry, into routine surveillance to improve detection speed and sensitivity, especially for multidrug-resistant fungi.

## 5. Conclusions

Airborne fungal pathogens pose significant and ongoing threats within healthcare environments, particularly to immunocompromised patients. Despite the availability of various sampling and analysis methods, the lack of standardized protocols hampers effective monitoring and control of fungal contamination. The integration of enhanced detection methods should be considered for improving detection speed and sensitivity, which is crucial for timely management of multidrug-resistant strains. Addressing environmental factors, alongside routine infection control practices, is essential to minimize fungal proliferation and exposure. Most importantly, there is a pressing need for internationally recognized monitoring standards and guidelines. Without these, preventable fungal infections will continue to occur each year, placing both patients and medical staff at an increased risk of infection.

## Figures and Tables

**Table 1:** Characteristics of included studies.

Citation	Country	Sampling Location	Fungi Detected	Guidelines/Recommendations
Ablola & Bungay (2020) [25]	Philippines	Three departments of surgery wards and three patient rooms	*Alternaria* *Aspergillus flavus* *Aspergillus fumigatus* *Aspergillus niger* *Curvularia* *Penicillium* *Rhizopus*	*Aspergillus* spp. HEPA-filtered air: <1 CFU/m^3^ Area with no air filtration: <5 CFU/m^3^
Alghamdi et al. (2023) [26]	Saudi Arabia	Pediatric intensive care unit	*Alternaria* *Aspergillus* *Chaetomium* *Cladosporium* *Curvularia* *Fusarium* *Helminthosporium* *Monilia* *Penicillium* *Rhizopus*	None
Aziz et al. (2024) [27]	Indonesia	Hematology ward, adult intensive care ward	*Aspergillus* *Aureobasidium* *Exophiala* *Geotrichum* *Mucor* *Penicillium* *Rhizopus* *Tricophyton* Yeasts	None
Buchanan et al. (2020) [24]	U.S.	Three mobile dust-containment carts with HEPA filtration in a hospital	*Aspergillus* *Cladosporium* *Curvularia* *Rhizopus*	None
Chen et al. (2024) [28]	China	Four specialized hospitals and one large hospital	*Alternaria* *Aspergillus ruber* Ascomycota *Cercospora* *Chromocleista* *Flammulina* *Fusarium* *Lacrymaria* *Mycosphaerella* *Pleurotus* *Wallemia*	None
Chen et al. (2022) [29]	Taiwan	Twelve negative pressure, 22 slightly negative pressure, 46 neutral pressure rooms, and 14 liver intensive care unit rooms	*Aspergillus fumigatus*	None
do Nascimento et al. (2023) [30]	Brazil	Twelve hospitals and medical clinics	*Candida glabrata* *Candida orthopsilosis* *Candida parapsilosis* *Cystobasidium slooffiae* *Fereydounia khargensis* *Hortaea werneckii* *Jaminaea lanaiensis* *Moniliella* *Papiliotrema flavescens* *Pseudozyma hubeiensis* *Pseudozyma siamensis* *Rhodotorula mucilaginosa* *Torulaspora delbrueckii* *Trichosporon mucoides*	None
Gorzynska et al. (2023) [31]	Poland	Patient rooms, bathrooms, treatment rooms, corridors	*Alternaria* *Aspergillus* spp. *Aspergillus fumigatus* *Cladosporium* *Fusarium* Mucormycetes ^a^ *Penicillium* Yeasts	None
Guvenir et al. (2023) [32]	Cyprus	Gynecology service rooms, cardiology service medicine preparation room, surgery service medicine preparation room, internal medicine room, geriatrics service medicine room, emergency room, drug preparation room, and operation rooms	*Aspergillus flavus*	Bioaerosols: ○ 0 CFU/m^3^ undetectable ○ 1–499 CFU/m^3^ low ○ 500–999 CFU/m^3^ medium ○ More than 1000 CFU/m^3^ high (European Commission report)
Ketabi et al. (2022) [33]	Iran	Three Operating Rooms and one ICU ward	*Aspergillus* Hyaline filamentous fungi *Mucor* *Penicillium* *Rhizopus* *Ulocladium*	None
Lemos et al. (2024) [34]	Brazil	Intensive care unit, medical clinic unit, and urgency and emergency unit	*Aspergillus flavus* *Aspergillus fumigatus* *Aspergillus nidulans* *Aspergillus niger* *Aspergillus terreus*	Lower than 750 CFU/m^3^ (Brazilian Ministry of Health and the National Health Surveillance Agency)
Mirhoseini et al. (2020) [35]	Iran	Cardiac care unit, neonatal intensive care unit, cancer blood ward, ENT (ear, nose, throat) operation room, and eye operation room	*Acremonium* *Alternaria* *Aspergillus flavus* *Aspergillus fumigatus* *Aspergillus niger* *Cladosporium* *Curvularia* *Drechslera* *Fusarium* *Mucor* *Paecilomyces* *Penicillium* *Rhizopus* *Stemphylium* *Trichoderma* *Trichothecium* Yeasts	None
Montazeri et al. (2020) [36]	Iran	Burns ward, derm ward, emergency department, and operating room	*Alternaria* *Aspergillus* ^b^ *Aspergillus flavus* *Aspergillus niger* *Cladosporium* *Mucor* *Penicillium* *Rhizopus*	Bioaerosols: ○ 0 CFU/m^3^ undetectable ○ 1–499 CFU/m^3^ low ○ 500–999 CFU/m^3^ medium ○ More than 1000 CFU/m^3^ high (European Commission report) Lower than 50 CFU/m^3^ in the hospital air (WHO)
Mori et al. (2020) [37]	Japan	Hematology ward of Keio University Hospital	*Alternaria* *Arthrinium* *Aspergillus* spp. *Aspergillus fumigatus* *Aspergillus niger* *Aspergillus sydowii* *Cladosporium* *Cryptococcus* *Monochaetia* *Penicillium* *Phoma* *Rhinocladiella* *Rhodotorula* *Wallemia*	None
Pedrosa et al. (2022) [38]	Brazil	Neonatal intensive care units and six operating rooms	*Acremonium* *Alternaria* *Aspergillus candidus* *Aspergillus deflectus* *Aspergillus granulosus* *Aspergillus niveus* *Aspergillus sclerotiorum* *Aspergillus ungüis* *Aureobasidium* *Bipolaris* *Candida* *Chrysosporium* *Cladosporium* *Curvularia* *Fusarium* *Geotrichum* *Monilia* Mycelia sterilia group *Paecilomyces* *Penicillium* *Scopulariopsis*	None
van Rhijn et al. (2021) [39]	United Kingdom	Cystic Fibrosis Centre	*Aspergillus fumigatus* *Aspergillus niger* *Geotrichum* *Penicillium*	None
Yerbanga et al. (2024) [40]	Burkina Faso	Infectious diseases ward, internal medicine ward, nephrology ward, pulmonology ward, medical emergency ward, and pediatric ward	*Aspergillus fumigatus*	None
Yousefzadeh et al. (2022) [41]	Iran	Men’s ward, women’s ward, lung, neurology, infectious, ICU, burn unit, operating room, emergency room	*Alternaria* *Aspergillus flavus* *Aspergillus niger* *Chrysosporium* *Cladosporium* *Cranosporium* *Geotrichum* *Ulocladium* *Penicillium* *Rhizopus* *Scopulariopsis* Yeasts	None

^a^ Mucoromycetes is a taxonomic class, which includes *Mucor* and *Rhizopus*, but the published study did not report a fungal genus. ^b^ Reported in the published study as *Aspergillus* CDV.

**Table 2:** Fungal concentration by healthcare facility area.

Citation	Healthcare Area	Fungal Concentration (CFU/m^3^)
Ablola & Bungay (2020) [25]	Non-air-conditioned wards Air-conditioned wards	73 151
Alghamdi et al. (2023) [26]	Hospital 1 Non-protective environment Protective environment Semi-protective environment Hospital 2 Non-protective environment Protective environment Semi-protective environment	<1 <1 2 <1 1 1
Aziz et al. (2024) [27]	Intensive care unit Hematology ward	17 237
Buchanan et al. (2020) [24]	Before construction Outside mobile dust containment cart (MDCC) HEPA exhaust from MDCC Inside MDCC	4 4 4 11
Chen et al. (2024) [28]	Not reported	Not reported
Chen et al. (2022) [29]	Liver intensive care unit rooms Neutral pressure rooms Negative pressure rooms Slightly negative pressure rooms	2 13 34 81
do Nascimento et al. (2023) [30]	Not reported	Not reported
Gorzynska et al. (2023) [31]	Hematology ward Transplant/post-transplant rooms Pediatric hematology ward	0 to 87 0 to 237 0 to 345
Guvenir et al. (2023) [32]	University Hospital	1 to 55
Ketabi et al. (2022) [33]	Before and after using HEPA filtration Operating theater 2 Operating theater 3 Operating theater 1 Intensive care unit	8 to 3 9 to 3 10 to 3 11 to 5
Lemos et al. (2024) [34]	Intensive, medical, and emergency units	224
Mirhoseini et al. (2020) [35]	Blood cancer ward Ear, nose, and throat operation room Eye operation room Neonatal intensive care unit Cardiac care unit	8 16 17 26 30
Montazeri et al. (2020) [36]	Operating theaters Emergency department Burn unit Between sections Derm ward	32 50 57 63 110
Mori et al. (2020) [37]	Not reported	Not reported
Pedrosa et al. (2022) [38]	Neonatal intensive care unit 1 Neonatal intensive care unit 3 Neonatal intensive care unit 2 Surgical center	78 108 126 140
van Rhijn et al. (2021) [39]	Not reported	Not reported
Yerbanga et al. (2024) [40]	Not reported	Not reported
Yousefzadeh et al. (2022) [41]	Operating room Burn ward Intensive care unit Internal men’s ward Infectious ward Emergency room Internal women’s ward Neurology ward Lung ward	<1 43 56 62 63 106 124 154 223

**Table 3:** Sampling and analysis methodology utilized in each of the included studies.

Citation	Culture Media	Impaction Sampler	Sampler Height	Flow Rate	Incubation Time/Temperature	Identification Methods
Ablola & Bungay (2020) [25]	Malt extract agar with chloramphenicol (MEAC)	Andersen six-stage sampler	— ^a^	28.3 L/min, 15 min	37 °C for 3–5 days	microscopic features
Alghamdi et al. (2023) [26]	Sabouraud Dextrose Agar (SDA)	SpinAir IUL	— ^a^	100 L/min	30 °C for 5–7 days	— ^a^
Aziz et al. (2024) [27]	SDA with Chloramphenicol	MAS-100NT Microbial Air Sampler	1 m	100 L/min	30 °C for 10 days	macroscopic and microscopic characteristics
Buchanan et al. (2020) [24]	Inhibitory mold agar	SAS Super 100, microbial air sampler	— ^a^	1000 L	Room temp. for 7 days	— ^a^
Chen et al. (2024) [28]	Glass microfiber filter media	atmospheric particulate samplers and ARA NFRM Sampler	1.5 m	100 L/min	— ^a^	PCR ^c^
Chen et al. (2022) [29]	Malachite green agar 2.5 ppm plates	MAS-100NT Microbial Air Sampler	~1.5 m	500 L total	25 °C for 7 days	morphological characteristics and MALDI-ToF ^b^
do Nascimento et al. (2023) [30]	Dichloran Rose-Bengal Chloramphenicol agar	Andersen single-stage sampler	1.5 m	28.3 L/min, 10 min	25 °C	PCR ^c^
Gorzynska et al. (2023) [31]	SDA with Chloramphenicol	MicroBio MB1 air sampler	1.2–1.4 m	100 L/min	25 °C for 2–10 days	macroscopic and microscopic characteristics
Guvenir et al. (2023) [32]	Columbia blood agar (OXOID) with 5% blood	IDEAL 3P device	— ^a^	— ^a^	37 °C for 48 h	— ^a^
Ketabi et al. (2022) [33]	SDA with Chloramphenicol	Andersen single-stage sampler	1.5 m	28.3 L/min, 2 min	32 °C for 7–10 days	macroscopic and microscopic characteristics
Lemos et al. (2024) [34]	Dichloran Rose-Bengal Chloramphenicol agar	MiniCapt Microbial Air Sampler	1.5 m	100 L/min	30 °C for 3 days	macroscopic and microscopic characteristics/PCR ^c^
Mirhoseini et al. (2020) [35]	MEAC	Andersen single-stage sampler	1.5 m	28 L/min, 5 min	27 °C for 3–7 days	macroscopic and microscopic characteristics
Montazeri et al. (2020) [36]	SDA with Chloramphenicol	Quick Take30 pump	— ^a^	28.3 L/min, 5 min	28–30 °C for 3–5 days	macroscopic and microscopic characteristics
Mori et al. (2020) [37]	Potato dextrose agar	BIOSAMP MBS-1000	— ^a^	50, 100, and 250 L	25 °C for 4–5 days and 2 days at room temp.	macroscopic and microscopic characteristics
Pedrosa et al. (2022) [38]	SDA with Chloramphenicol	Andersen single-stage sampler	1.5	28.3 L/min, 10 min	25 °C for 7 days	macroscopic and microscopic characteristics
van Rhijn et al. (2021) [39]	MEA	SAS	1.2–1.5 m	1 m^3^ air over 10 min	30 °C for 4 days	macroscopic and microscopic characteristics
Yerbanga et al. (2024) [40]	SDA with Chloramphenicol	SpinAir	1.5 m	100 L/min	30 °C for 2–7 days	microscopic features
Yousefzadeh et al. (2022) [41]	MEAC	Andersen single-stage sampler	1–1.5 m	28.3 L/min, 5 min	25–27 °C for 3 days	macroscopic and microscopic characteristics

^a^ Not reported; ^b^ Matrix-assisted laser desorption/ionization time of flight mass spectrometry; ^c^ Polymerase chain reaction.

**Table 4:** Fungal concentrations reported in the included studies.

Citation	Species	Concentration (CFU/m^3^)
Ablola & Bungay (2020) [25]	*Alternaria* *Aspergillus flavus* *Curvularia* *Penicillium* *Rhizopus* *Aspergillus fumigatus* *Aspergillus niger*	0 to 1 0 to 53 1 to 6 1 to 14 1 to 14 8 to 255 18 to 49
Aziz et al. (2024) [27]	*Geotrichum* *Aureobasidium* *Mucor* *Trichophyton* *Exophiala* *Penicillium* *Aspergillus* spp. Yeasts	9 15 23 31 36 86 89 137
Gorzynska et al. (2023) [31]	*Alternaria* *Aspergillus* spp. *Aspergillus fumigatus* *Penicillium* *Cladosporium*	0 to 10 0 to 13 0 to 13 0 to 20 0 to 131
Lemos et al. (2024) [34]	*Aspergillus* spp.	17 to 30
Yerbanga et al. (2024) [40]	*Aspergillus fumigatus*	1 to 40

**Table 5:** Recommendations for air sampling best practices.

Practice	Recommendation
Sampler Type	Impaction air sampler
Sampler Placement	1 to 1.5 m above the floor and at least one meter from walls/obstacles
Sampling Location	General patient areas, immunocompromised patient areas, and rooms/corridors impacted by construction
Flow Rate	28.3 to 100 L/min
Air Volume	50 to 1000 L
Sampling Duration	5 to 10 min
Culture Media	Consider target fungi; SDA and MEA with chloramphenicol are optimal choices
Sample Collection	Duplicate samples to be incubated at different temperatures; include outdoor reference samples
Incubation Time/Temperature	25 °C for 3 to 5 days and 37 °C for 2 days

## Data Availability

Data are contained within the article.

## Abbreviations

The following abbreviations are used in this manuscript:

CFU	Colony Forming Unit
HEPA	High-Efficiency Particulate Air
WHO	World Health Organization
U.S.	United States
HIV/AIDS	Human immunodeficiency virus/acquired immunodeficiency syndrome
PRISMA	Preferred Reporting Items for Systematic Review and Meta-Analysis
PCR	Polymerase Chain Reaction
HVAC	Heating, Ventilation, and Air Conditioning
MALDI-ToF	Matrix-Assisted Laser Desorption/Ionization Time-of-Flight
DNA	Deoxyribonucleic Acid
MEAC	Malt Extract Agar with Chloramphenicol
SDA	Sabouraud Dextrose Agar
MEA	Malt Extract Agar
ICU	Intensive Care Unit
MDCC	Mobile Dust-Containment Cart
SARS-CoV-2	Severe Acute Respiratory Syndrome Coronavirus 2
CDC	Centers for Disease Control and Prevention
OSHA	Occupational Safety and Health Administration
ENT	Ear, nose, and throat

## Appendix A

**Table A1:** Critical appraisal of included cross-sectional studies.

First Author (Year)	Q1	Q2	Q3	Q4	Q5	Q6	Q7	Q8	Score
Ablola et al. (2020) [25]	Y	Y	Y	Y	Y	N	Y	Y	14/16
Alghamdi et al. (2023) [26]	Y	Y	Y	Y	Y	U	Y	Y	15/16
Aziz et al. (2024) [27]	Y	Y	Y	Y	Y	N	Y	Y	14/16
Chen et al. (2022) [29]	Y	Y	Y	Y	Y	U	Y	Y	15/16
Chen et al. (2024) [28]	Y	Y	Y	Y	Y	U	Y	Y	15/16
Nascimento et al. (2023) [30]	Y	Y	Y	Y	Y	N	Y	Y	14/16
Gorzynska et al. (2023) [31]	Y	Y	Y	Y	Y	N	Y	Y	14/16
Guvenir et al. (2023) [32]	Y	Y	Y	Y	Y	N	Y	Y	14/16
Lemos et al. (2024) [34]	Y	Y	Y	Y	Y	N	Y	Y	14/16
Mirhoseini et al. (2020) [35]	Y	Y	Y	Y	Y	N	Y	Y	14/16
Montazeri et al. (2020) [36]	Y	Y	Y	Y	Y	Y	Y	Y	16/16
Mori et al. (2020) [37]	Y	Y	Y	Y	Y	N	Y	Y	14/16
Pedrosa et al. (2022) [38]	Y	Y	Y	Y	U	N	Y	Y	13/16
Yerbanga et al. (2024) [40]	Y	Y	Y	Y	U	N	Y	Y	13/16
Yousefzadeh et al. (2022) [41]	Y	Y	Y	Y	Y	U	Y	Y	15/16

JBI Critical Appraisal Checklist for Cross-sectional studies: Q1: Were the criteria for inclusion in the sample clearly defined? Q2: Were the study subjects and the setting described in detail? Q3: Was the exposure measured in a valid and reliable way? Q4: Were objective, standard criteria used for measurement of the condition? Q5: Were confounding factors identified? Q6: Were strategies to deal with confounding factors stated? Q7: Were the outcomes measured in a valid and reliable way? Q8: Was appropriate statistical analysis used? Y: Yes (2 points); N: No (0 points); U: Unclear (1 point). Scores for risk of bias: Low: 13–16, Moderate: 9–12, High: below 9.

**Table A2:** Critical appraisal of included cohort studies.

First Author (Year)	Q1	Q2	Q3	Q4	Q5	Q6	Q7	Q8	Q9	Q10	Q11	Score
Van Rhijn et al. (2021) [39]	Y	Y	Y	Y	Y	N/A	Y	Y	N/A	N/A	Y	16/16 *

JBI Critical Appraisal Checklist for Cohort Studies: Q1: Were the two groups similar and recruited from the same population? Q2: Were the exposures measured similarly to assign people to both exposed and unexposed groups? Q3: Was the exposure measured in a valid and reliable way? Q4: Were confounding factors identified? Q5: Were strategies to deal with confounding factors stated? Q6: Were the groups/participants free of the outcome at the start of the study (or at the moment of exposure)? Q7: Were the outcomes measured in a valid and reliable way? Q8: Was the follow up time reported and sufficient to be long enough for outcomes to occur? Q9: Was follow up complete, and if not, were the reasons to loss to follow up described and explored? Q10: Were strategies to address incomplete follow up utilized? Q11: Was appropriate statistical analysis used? * Total possible points and scores for risk of bias were adjusted to accommodate the N/A answers. Y: Yes (2 points); N: No (0 points); U: Unclear (1 point). Scores for risk of bias: Low: 13 or higher, Moderate: 9–12, High: below 9.

**Table A3:** Critical appraisal of included quasi-experimental studies.

First Author (Year)	Q1	Q2	Q3	Q4	Q5	Q6	Q7	Q8	Q9	Score
Buchanan et al. (2020) [24]	Y	Y	U	N/A	N	Y	Y	N/A	N	9/14 *
Ketabi et al. (2022) [33]	Y	Y	Y	N/A	Y	Y	Y	N/A	Y	14/14 *

JBI Critical Appraisal Checklist for Cross-sectional studies: Q1: Is it clear in the study what is the “cause” and what is the “effect”? Q2: Was there a control group? Q3: Were participants included in any comparisons similar? Q4: Were the participants included in any comparisons receiving similar treatment/care, other than the exposure or intervention of interest? Q5: Were there multiple measurements of the outcome, both pre and post the intervention/exposure? Q6: Were the outcomes of participants included in any comparisons measured in the same way? Q7: Were outcomes measured in a reliable way? Q8: Was follow-up complete and if not, were differences between groups in terms of their follow-up adequately described and analyzed? Q9: Was appropriate statistical analysis used? * Total possible points and scores for risk of bias were adjusted to accommodate the N/A answers. Y: Yes (2 points); N: No (0 points); U: Unclear (1 point). Scores for risk of bias: Low: 12 or higher, Moderate: 9–11, High: below 9.
